# Retrospective analysis of criteria for oncological completion surgery of neuroendocrine tumors of the appendix

**DOI:** 10.1007/s00423-024-03603-6

**Published:** 2025-01-20

**Authors:** Sabine Wächter, Dimitrios Panidis, Moritz Jesinghaus, Anja Rinke, Monika Heinzel-Gutenbrunner, Elisabeth Maurer, Detlef K. Bartsch

**Affiliations:** 1 Department of Visceral, Thoracic and Vascular Surgery, Philipps-Universität Marburg, Baldingerstraße, Marburg, 35043 Germany; 2 Institute of Pathology, Philipps-Universität Marburg, Marburg, Baldingerstraße, 35043 Germany; 3 Department of Gastroenterology, Philipps-Universität Marburg, Marburg, Baldingerstraße, 35043 Germany; 4 MH Statistik Beratung, Bienenweg 8, Marburg, 35041 Germany

**Keywords:** Neuroendocrine tumor, Appendix, Oncological completion resection, Hemicolectomy, Lymphnode metastases, Tumor size

## Abstract

**Purpose:**

Neuroendocrine neoplasms of the appendix (aNET) are rare tumors that are often diagnosed by pathology as an incidental finding after appendectomy for acute appendicitis. Several guidelines proposed risk criteria to indicate oncological completion surgery after appendectomy. The aim of this study was to evaluate the reliability of proposed criteria for completion surgery of aNET.

**Methods:**

Patients with aNET treated at ENETS center of excellence Marburg between 2002 and 2022 were retrieved from a prospective data base. Demographic data, histopathological findings, including formerly proposed criteria to indicate oncological completion surgery, histological results of the completion resection and disease-free survival were evaluated.

**Results:**

82 patients with a median age of 35 (range 8–82) years were analysed. 72 (88%) patients underwent an emergency appendectomy because of acute appendicitis. 11 (13%) patients received an ileocecal resection or right hemicolectomy. Seven (8.5%) patients had lymph node metastases and three (3.6%) patients had distant metastases at the initial operation. 27 (33%) patients underwent completion surgery by right hemicolectomy according to guideline criteria, but postoperative histology detected lymph node and distant metastases in only six (22%) and zero patients resulting in an overtreatment of 21 (75%) patients. A tumor size of > 2 cm was the only significant criterion which was associated with lymph node metastases (*p* < 0.05). After a median follow-up of 62 months (range 2-264) 76 (96%) of the patients in stages I to III were alive with no evidence of disease.

**Conclusion:**

aNET have an excellent prognosis in stages I-III and distant metastases are rare. Formerly proposed criteria for oncological completion surgery have to be adopted and discussed for every patient, as they might result in an overtreatment in at least 75% of patients.

## Introduction

Neuroendocrine tumors of the appendix (aNET) account for 50–77% of all appendiceal neoplasms and are usually discovered incidentally after appendectomy for acute appendicitis or after laparotomy for other reasons [1]. In the vast majority aNET are well differentiated low grade (> 80% grade 1) neoplasms and rarely associated with a secretory syndrome [1, 2]. A number of histopathological parameters have been proposed in guidelines of several national and international societies such as ENETS, NANETS, UKINETS to indicate oncological completion surgery, since these parameters were associated with an increased risk of regional lymph node involvement and an increased risk of recurrence [3, 4]. These histopathological criteria include a tumor size > 2 cm, infiltration of the mesoappendix > 3 mm, angioinvasion, grading 2 or 3 and R1/R2 resection, respectively. The results of a previous European retrospective multicenter study, which included 278 patients with aNET of 1–2 cm in size who either had an appendectomy (*n* = 168, 59%) or right hemicolectomy (*n* = 115, 41%), questioned these criteria and the resulting recommendations [5]. After a median follow-up of 13 years the overall survival was similar between patients with appendectomy and right hemicolectomy (HR 0.88 [95% CI 0.36–2.17]; *p* = 0.71). Thus, recently updated ENETS guidelines [6] recommended oncological completion surgery only in a tumor size > 2 cm and in case of incomplete resection (R1 or R2). In case of a tumor size between 1–2 cm certain risk factors, including high grade aNET, angioinvasion, lymphatic invasion and individual patient's expectations may justify oncologic completion surgery. There was a lack of evidence as to whether completion surgery is of any benefit in the presence of serosal perforation. The guidelines of several societies (ENETS, NANETS, DGAV) advocate oncologic right hemicolectomy as the procedure of choice in patients with aNET fulfilling histological criteria for completion surgery [3, 4, 7]. Since this procedure can be associated with short- and long-term postoperative morbidity, along with long-term health-related quality of life impairment [8, 9] ileocecal resection has also been applied in some patients, but mainly in children [10, 11]. At present the indication for either ileocecal resection or right hemicolectomy in aNET remains unclear, in any case resection of the ileocecal vessels at their origin is mandatory [8]. The aim of the present study was to evaluate the reliability of proposed histopathological criteria to indicate oncologic completion surgery for aNET.

## Methods

Patients with aNET treated at the ENETS Center of Excellence Marburg between June 2002 and December 2022 were retrieved from a prospective database. Goblet cell carcinomas were excluded. Demographic data, histopathological findings, including the criteria for oncologic completion surgery such as tumor size > 2 cm, infiltration of the mesoappendix > 3 mm, angioinvasion, grading (G) 2/3 and R1/R2 resection, were retrospectively evaluated by analysing the original pathology reports and available pathological slides by an experienced pathologist (MR). Tumor grading was determined based on the Ki-67 index, with the following thresholds: Ki-67 ≤ 2% was classified as G1, Ki-67 between 2% and 20% as G2, and Ki-67 > 20% as G3. TNM staging was performed according to ENETS classification [6]. The patients, who initially underwent external surgery, the resection specimens and histological findings were requested and, if available, re-evaluated.

Histological results after completion surgery were analysed for residual disease, the presence of lymph node and distant metastases. Follow-up with regard to tumor recurrence, development of metastases, disease-free and overall survival, if indicated, was performed by the ENETS CoE Marburg or by the personal physician. Follow-up imaging included physical examination, ultrasonography of the abdomen, MRI of the abdomen and functional SRS-imaging every 6–12 months. The determination follow-up date was March 1, 2023. Since no follow-up examination was recommended in stage I according to guidelines, all patients with stage I who had no documented disease recurrence by either their personal physician, the CoE Marburg or death certificate were classified as patients with no evidence of disease.

The standard completion surgery was conventional oncologic right hemicolectomy. The majority of the patients (26/27, 96%) had an open surgical procedure, whereas only one patient (1/27, 4%) was operated laparoscopically. Clinically relevant complications after the oncological completion surgery were defined according to Clavien-Dindo [12]. The study was approved by the Marburg Ethics Committee (No. 23-259RS).

Continuous variables are expressed as medians with interquartile ranges (IQR), and categorical variables are presented as a proportion. Potential predictors of lymph node metastases were tested simultaneously by binary logistic regression analysis and the corresponding odds ratio (OR) including 95% confidence interval (95%-CI) and p-values were calculated. The level of statistical significance was set at α = 0.05.

## Results

98 patients with aNET were identified who were treated by surgery at the CoE Marburg. In 16 of those patients, who were operated in outside hospitals, sufficient information regarding pathological findings and follow-up could not be obtained. Thus, these patients were excluded from the study and a total of 82 patients with aNET remained for analysis. The median age of the patients was 35 years (range 8–82), only 13 (16%) patients were younger than 18 years. 51 (62%) patients were women and 31 (38%) patients were men.

The initial operation in 71 (87%) patients was an emergency appendectomy for acute appendicitis. In 5 (6%) patients the initial operation was an ileocecal resection because of an unclear stenosis of the terminal ileum (*n* = 1), terminal ileitis with an incidental finding of an aNET (*n* = 1) or because of a perforated appendicitis with perityphlitic abscess (*n* = 1). Another patient was operated for acute appendicitis, but intraoperatively an aNET with peritoneal carcinomatosis was found. The fifth patient showed a tumor in the cecum on MRI and underwent elective ileocecal resection.

In 6 (7%) patients the initial operation was a right hemicolectomy for the following reasons: in 2 patients an aNET > 2cm was found during colonoscopy and therefore both underwent primary right hemicolectomy; two patients underwent elective hemicolectomy for adenocarcinoma of the ascending colon with an incidental finding of an aNET and one patient underwent emergency hemicolectomy for a volvulus with an incidental finding of aNET, respectively. The sixth patient underwent emergency surgery for suspected appendicitis, but intraoperatively an aNET with peritoneal carcinomatosis was detected. In all evaluated patients, the histopathological diagnosis of aNET was confirmed postoperatively. Only in two patients the diagnosis was identified preoperatively via colonoscopy. The characteristics of patients with aNET at initial surgery were summarized in Table1.

**Table 1:** Clinical characteristics of aNET at initial operation

Parameter	N
No. of patients	82
Median age in years (range)	35 (8–82)
Pat. < 18 years (%)	13 (16%)
Male/Female (%)	31 (38%)/51 (62%)
Pat. with emergency operations for appendicitis (%)	72 (88%)
Pat. with initial appendectomy (%)	71 (87%)
Pat. with initial ileocecal resection (%)	5 (6%)
Pat. with initial right hemicolectomy (%)	6 (7%)
Pat. with distant metastases at initial operation (%)	3/82 (3.6%)
Pat. with oncological completion surgery (%)	27/82 (33%)
Pat.with lymph node metastases after completion surgery (%)	7/82 (8.5%)

*No*-number, *Pat-*patients

Histopathological analysis described a tumor size < 1cm in 43 (52%) patients, a size 1-2cm in 29 (35%) patients and a size > 2cm in 10 (13%) patients. 72 (87.9%) patients had a tumor grading G1, nine (10.9%) patients G2 and only one (1.2%) patient G3, respectively. Vascular invasion was present in three (4%) patients. There was a perineural invasion in five (6%) patients and mesoappendix invasion > 3 mm in 23 (28%) patients. The resection status was R0 in 75 (91%) patients, R1 in four (5%) patients and R2 in three (4%) patients (Table 2). All R1-resected patients were in a post-appendectomy state and subsequently underwent a right hemicolectomy according to ENETs guidelines achieving a N0 and R0 status, so that none of these patients received adjuvant chemotherapy. According to the ENETS classification [6] 22 (27%) patients had stage I disease, 50 (61%) patients stage II, seven (8%) patients stage III and three (4%) patients stage IV disease, respectively. Of the 82 patients included in this study, 59 had the tumor located at the tip of the appendix, seven near the base. In the remaining 16 patients, the exact location was not mentioned in the pathology report and was unknown.

**Table 2:** Histological characteristics of aNET after initial surgery (*n* = 82)

Parameter	No. patients
Tumor size < 1cm (%) 1-2cm (%) > 2cm (%)	43/82 (52%) 29/82 (35%) 10/82 (13%)
G1 (%) G2 (%) G3 (%)	72/82 (87.9%) 9/82 (10.9%) 1/82 (1.2%)
Vascular invasion (%)	3/82 (4%)
Perineural invasion (%)	5/82 (6%)
Invasion of the mesoappendix > 3mm (%)	23/82 (28%)
Resection status R0 (%) R1 (%) R2 (%)	75/82 (91%) 4 /82 (5%) 3/82 (4%)
ENETS stage I (%) II (%) III (%) IV (%)	22/82 (27%) 50/82 (61%) 7/82 (8%) 3/82 (4%)
No. of patients with indicated right hemicolectomy according to ENETS criteria [4] (%)	25/82 (30.4%)
No. of patients with right hemicolectomy because of fulfilled ENETS criteria (%)	24/25 (96%)
No. of patients with right hemicolectomy without fulfilling the ENETS (%)	3/57 (5.2%)
No. of patients without right hemicolectomy although ENETS criteria were fulfilled (%)	1/25 (4%)

*No*-number

Oncologic completion surgery by right hemicolectomy was indicated by the tumor board in 25 (30.4%) patients, primary according to guideline recommendations [3, 6, 7] Completion right hemicolectomy was performed in 24 of these patients, one patient was not reoperated because of a poor general condition. In addition, there were three patients who had a reoperation, although the criteria for completion surgery were not fulfilled. These patients were operated between 2005–2013 in external hospitals (before the existence of the ENETS Guidelines). Thus, overall 27 (33%) patients underwent completion right hemicolectomy (Table 3). The tumor sizes of these patients were < 1 cm, 1–2 cm and > 2 cm in three (11%), 16 (59%) and eight (30%) patients, respectively. Four (15%) patients had a R1 resection. Additional histological parameters to indicate oncologic completion surgery were infiltration of the mesoappendix > 3 mm in 20 (74%) patients, grading G2 in six (22%) patients, perineural infiltration in three (11%) patients and vascular infiltration in two (7%) patients, respectively (Table 3).

**Table 3:** Characteristics of patients in whom completion surgery was indicated

Parameters at initial OP	Patients with completion surgery (*n* = 27)	Patient with LN mets. at completion surgery (*n* = 6/27, 22%)	Odds-Ratio	95%-CI	*p*-Value
Tumor size > 2cm (%)	8/27 (30%)	5/8 (62.5%)	23.1	1.2–478.6	0.04*
Mesoappendiceal Infiltration > 3mm (%)	20/27 (74%)	6/20 (30%)			1
Vascular infiltration (%)	2/27 (7%)	0/2 (0%)			1
Perineural infiltration (%)	3/27 (11%)	1/3 (33.3%)	0.19	0.01–6.29	0.35
R1/R2 (%)	4/27 (15%)	0/4 (0%)			1
Grading G2 (%)	6/27 (22%)	1/6 (17%)	0.51	0.02–15.86	0.7

*CI *= confidence interval *statistically significant

Histopathological work-up of the completion resection specimens showed residual disease in one of 27 (3.7%) patients and lymph node metastases in six of 27 (22.2%) patients. Median 21 (13–48) lymph nodes were dissected. None of the patients with a tumor < 1 cm had lymph node metastases compared to one of 16 (6.3%) patients with a tumor 1–2 cm and five of eight (62.5%) patients with tumor > 2 cm. This was also true for six of 20 (30%) patients with infiltration of the mesoappendix > 3 mm, but five of those had also tumors > 2 cm. One of three patients with perineural vessel infiltration and one of six patients with G2 grading had lymph node metastases (Table 3/4). None of the 27 patients had distant metastases at the time of completion right hemicolectomy. Thus, the only significant parameter associated with the presence of lymph node metastases was a tumor size > 2 cm (*p* = 0.04). The remaining risk factors had no significant association with lymph node metastases (*p* > 0.05)

None of the 27 patients with completion surgery had clinically relevant complications (CD ≥ 3) after completion right hemicolectomy. Four of 27 patients had minor complications like superficial wound infections, postoperative gastrointestinal atony and incisional hernias (Table 4).

**Table 4:** Parameters after reoperation

Parameters after reoperation	Patients with completion surgery (*n* = 27)	Patient with LN mets. at completion surgery (*n* = 6/27, 22%)
resected lymph nodes, median (range)	21 (13–48)	17 (13–48)
residual disease in specimen (%)	1/27 (3,7%)	1/6 (18,7%)
postop. complications (CD ≥ 3) (%)	0/27 (0%)	0/6 (0%)

*LN*- lymph nodes, *postop*-postoperative

Three patients had stage IV disease with liver metastases and peritoneal carcinomatosis at initial surgery. These patients did not undergo oncologic completion surgery, because of a palliative situation with unresectable diffuse metastatic disease. The ages of these patients (two female, one male) were 31, 61 and 63 years at initial diagnosis. In one of these stage IV patients the tumor size was 1–2 cm, in two patients > 2 cm. The grading was G1, G2 and G3 in one patient each and all three patients had an infiltration of the mesoappendix > 3 mm. In two patients the initial surgery was an ileocecal resection and in one patient a right hemicolectomy. Postoperatively, all three patients had palliative treatment with somatostatin analogues. Two patients had additional peptid receptor radioligand therapy (PRRT), one patient chemotherapy with carboplatin and etoposid and target therapy with somatostatin analogues. At the last follow-up two patients were deceased 56 and eight months after initial surgery and the third patient was alive with disease 190 months after initial surgery.

Overall, after a median follow-up of 64 (2–264) months 21 of 22 (95%) patients with ENETS stage I, 48 of 50 (96%) patients with stage II, and all seven (100%) patients with stage III had no evidence of disease. One patient with stage I and two patients with stage II disease died to unrelated causes (Table 5). Overall, 76 (92.8%) patients remained free of disease, two patients with stage IV disease (2.4%) deceased tumor related, one patient with stage IV disease is alive with metastatic tumor (1.2%).

**Table 5:** Outcome

ENETS Stage [4]	median follow-up, months (range)	NED (%)	AWD (%)	DOD (%)	DURC (%)
I (*n* = 22)	64 (10–264)	21 (95%)	0 (0%)	0 (0%)	1 (5%)
II (*n* = 50)	60 (2–264)	48 (96%)	0 (0%)	0 (0%)	2 (4%)
III (*n* = 7)	53 (18–156)	7 (100%)	0 (0%)	0 (0%)	0 (0%)
IV (*n* = 3)	134 (39–190)	0 (0%)	1	2	-
all stages (*n* = 82)	64 (2–264)	76 (92.8%)	1 (1.2%)	2 (2.4%)	3 (3.6%)

*NED* no evidence of disease, *AWD* alive with disease, *DOD* dead of disease, *DURC* dead of unrelated cause

## Discussion

The demographic data of patients with aNET in the present study are consistent with the literature. The tumors occurred more frequently in women than in men and patients were median 35 years old at diagnosis [4]. As in other studies only less than 20% of patients were children or adolescents [4]. In the present study 88% of aNET were diagnosed incidentially after emergency appendectomy for acute appendicitis, which is approximately 80% in other studies [4]. Patients with aNET who initially had right hemicolectomy were either patients who had an aNET > 2 cm preoperatively diagnosed by colonoscopy or CT/MRI of the abdomen or had a colon carcinoma and the aNET was an incidental finding.

According to ENETS, NANETS, UKINETS and German guidelines a tumor size > 2 cm is the most important criterion to indicate oncologic completion surgery [3, 6, 7, 13]. This is supported by the present study, since at initial surgery seven of ten patients with aNET > 2 cm had lymph node metastases and three patients also had liver and peritoneal metastases. In addition, five of eight patients with oncologic completion surgery for a aNET > 2 cm had lymph node metastases. None of above mentioned guidelines recommend completion surgery in aNETs < 1 cm, since metastases are an extremely rare event as in the present study.

These recommendations are different for aNET sized between 1–2 cm. The percentage of lymph node metastases in this group after completion right hemicolectomy has been reported between 20% and 38% [4, 5, 14, 15], which is higher than 6.3% in the present study. In these studies mesoappendiceal invasion > 3 mm was observed in 4–43% of the evaluated patients, which was considered as an indication for completion surgery [3, 6, 7, 13]. After multivariate analysis mesoappendiceael invasion was associated with lymph node metastases in only one of three studies [15–17]. In our patient collective mesoappendiceal invasion > 3 mm was found in 28% of the evaluated patients. Only one of 18 patients with an aNET 1–2 cm and mesoappendiceal invasion > 3 mm had lymph node metastases after completion surgery. Overall, infiltration of the mesoappendix was present in six patients with lymph node metastases (30%) of the present series, but five of these patients had a tumor > 2 cm. Accordingly, based on our data, infiltration of the mesoappendix does not represent an independent risk factor for the presence of lymph node metastases.

Consistent with the data available in the literature, 5% of the patients we evaluated had an R1 resection during the primary surgery. A major reason for these R1 resections is that a large proportion of aNETS are located at the base of the appendix [4]. Neither in the present study nor in other studies R1 resection was associated with the presence of lymph node metastases [18]. R1 resection, however, is considered by all guidelines [3, 6, 7, 13] as an indication for oncological completion surgery to eliminate residual disease.

Vascular invasion in aNET 1–2 cm was described in 7–17% of cases [5, 18, 19], which was higher than the 4% prevalence in the present series. Vessel invasion, however, was not associated with lymph node metastases in the present series, nor in two recent multivariate analyses [18, 19].

The UKINETS guideline is the only one which includes perineural invasion as a risk factor to indicate completion surgery in aNET 1–2 cm [4]. Perineural invasion was noted in 6% of the present series and was not associated with lymph node metastases, which is in contrast to one previous study [18].

As in the present study (87.9%) the majority of aNET are G1 tumors (82–94%), the rest are either G2 (5–12%) or very rarely G3 (0.2 to 1%) lesions [5, 16, 18, 20–23]. In most of these previous studies no significant association between G2 or G3 grading and the presence of lymph node metastases could be established, which is most likely caused by the relatively low number of patients with G2/G3 aNET analysed. Furthermore, it is unknown to what extent – similar to what is suggested for pancreatic NET – a subclassification of G2 tumors into low- and high-grade tumors plays a significant role in aNET. This interesting question should be further investigated in lager studies [24].

The presented data and all previously published studies indicate that a tumor size > 2 cm is the only consistent risk factor that is associated with lymph node metastases, whereas all other proposed risk factors are not really verified. In the present study 78% of patients underwent completion surgery based on these risk factors and had no lymph node metastases. This fact raises concerns, whether the presence of histopathological risk factors others than a tumor size > 2 cm should be an indication for completion surgery.

Further indirect data can be extrapolated from studies, where patients fulfilled the criteria for oncologic completion surgery but did not undergo the procedure, support this concern. A recent meta-analysis, including 120 of 958 children with aNET, who fulfilled the proposed criteria for oncologic completion surgery, but had only appendectomy, revealed no recurrence or metastases after a median follow-up of 4.8 years [25]. In addition, a recent European multicenter study analysed 278 aNET patients with tumor size of 1–2 cm who fulfilled at least one of the proposed risk factors for oncologic completion surgery as defined in the 2016 ENETS guidelines [4] demonstrated that the long-term survivals were similar in patients who underwent only appendectomy (*n* = 163) compared to those who had completion right hemicolectomy. It was calculated that patients with appendectomy had a rate of 12.8% of lymph node metastases. This seemed not to be clinically relevant, since none of these patients developed clinically obvious metastases during a median follow-up of 13 years. The presented data and the afore mentioned studies support the new recommendation of the recently published ENETS guidance paper [6]. Oncologic completion surgery should be indicated in aNET > 2 cm and incomplete (R1/R2) appendectomies. In resected aNET between 1–2 cm the presence of the previously proposed risk factors such as mesoappendiceal invasion > 3 mm and high grade aNET are not a general indication for oncologic completion surgery. In aNET 1–2 cm completion surgery might be justified according to individual patient's choice after detailed information.

The guidelines of several societies (ENETS, NANETS, UKINETS, DGVS Leitlinie) recommend right hemicolectomy as the procedure of choice for oncologic completion surgery in patients with aNET as it was performed in all 27 patients of the present series [3, 6, 7, 13]. There is an ongoing debate about whether ileocecal resection, which may cause fewer postoperative morbidity than a hemicolectomy, could be a safe alternative [2, 3, 8]. To date no direct comparison exists between the two procedures, but in both procedures resection of the ileocecal artery at its origin from the superior mesenteric artery is mandatory. Ileocecal resection either as first line surgery or as completion surgery for aNET has been applied in some pediatric patients [8, 19]. One might extrapolate the results of a recent North American study of small intestine neuroendocrine tumors [9]. Patients who underwent oncologic right hemicolectomy had more resected lymph nodes than those with ileocecal resection (median 18 vs. 14, *p* = 0.004), but the oncological long-term outcome was similar. There was no difference in the incidence and severity of postoperative morbidity (both *p* > 0.05). In the present series the prevalence of postoperative clinically relevant complications after completion right hemicolectomy was zero. The patients we evaluated were exclusively resected using right hemicolectomy, so we are unable to comment on the role that ileocecal resection would have in this situation.

Thus future studies need to evaluate whether ileocecal resection for completion surgery in aNET is oncologically adequate.

Given that 3.6% of patients in our studied cohort already hat metastatic disease at initial diagnosis and that guidelines recommend further resection only for tumors > 2 cm, it is important to consider the recommended follow-up examinations for aNETS. Unfortunately, there is limited data regarding the appropriate duration of follow-up for patients with aNETs. According to the ENETS guidelines, follow-up is not necessary for patients with a N0 and/or R0 status following hemicolectomy, as well as for those with incidental aNETs after appendectomy without risk factors. Our study supports this recommendation, as none of these patients in our cohort experienced a recurrence. For patients with lymph node metastases after right hemicolectomy, follow-up every 6–12 months is recommended; however, there are no specific data on the required duration of follow-up [6]. In our study, none of the six patients with lymph node metastases experienced tumor recurrence during their follow-up examinations, suggesting no clear benefit of follow-up in this group (though the sample size is very small with only six patients).

Our study has some limitations. First, the retrospective nature of our study is a limitation which induced some potential bias. Second, although the majority of aNET were re-evaluated by an experienced pathologist, this was not possible for all specimens, and thus few histopathological risk factors could not be obtained. Third, patients with aNET < 1 cm had no regular follow-up to detect recurrent diseases, since this was not recommended in guidelines [6]. Fourth, despite being a ENETS center of excellence the study cohort is relatively small due to the low incidence of aNET.

## Conclusion

In conclusion the present study supports the recommendation for oncologic completion surgery in aNET > 2 cm, but not in aNET < 2 cm completely resected by appendectomy. In resected aNET sized 1–2 cm with certain risk factors, including mesoappendiceal invasion and high-grade tumors, completion surgery might be indicated according to patients‘ choice after detailled counselling.

## Data Availability

No datasets were generated or analysed during the current study.
